# Shared neural signatures in Functional Neurological Disorder and Chronic Pain: a multimodal narrative review

**DOI:** 10.1136/bmjno-2025-001032

**Published:** 2025-07-13

**Authors:** Siddarth Kannan, Kajal Patel, Daniela Di Basilio, Antonia Kirkby, Manoj Sivan, Anthony Jones, Rajiv Mohanraj, Abhijit Das

**Affiliations:** 1 University of Central Lancashire, Preston, UK; 2 Department of Neurology, Cleveland Clinic, Cleveland, USA; 3 Lancaster University, Lancaster, England, UK; 4 Department of Clinical Neuropsychology, North Staffordshire Combined Healthcare NHS Trust, Stoke-on-Trent, UK; 5 Academic Department of Rehabilitation Medicine, University of Leeds, Leeds, UK; 6 The University of Manchester, Manchester, England, UK; 7 Department of Neurology, The University of Manchester, Manchester, England, UK; 8 Neurology, Lancashire Teaching Hospitals NHS Foundation Trust, Preston, UK

**Keywords:** FUNCTIONAL NEUROLOGICAL DISORDER, PAIN, BRAIN MAPPING, PET, EEG

## Abstract

**Background:**

Functional neurological disorder (FND) frequently co-exists with chronic pain (CP), notably nociceptive and nociplastic (primary) pain disorders. The considerable overlap implies shared underlying mechanisms because of their similar clinical and epidemiological profiles. Although standard neuroimaging and electrophysiological tests typically show normal results in both FND and primary pain disorders, recent advancements in neuroimaging techniques have begun identifying neural biomarkers common to both conditions, though these findings remain preliminary and require further exploration.

**Method:**

We performed a detailed literature review of studies investigating neural activity in FND and chronic pain using electroencephalogram, magneto-encephalography, functional MRI, positron emission tomography and single photon emission computed tomography. Given the diverse nature of the reviewed studies, the synthesis is presented narratively.

**Results:**

Despite methodological differences, convergent data suggest disrupted neural networks across both FND and CP. Common findings include (1) hyperactivation of sensorimotor networks, (2) altered activity within the default mode network—a critical region for self-referential thought—and (3) dysfunction in emotional processing regions, notably the anterior cingulate cortex and insula. Thalamocortical dysrhythmia was identified as a potential unifying concept, characterised by abnormal theta and beta oscillations that enhance pain perception in CP and trigger functional symptoms in FND. Both conditions also exhibit reduced alpha oscillations, likely amplifying sensory sensitivity and emotional responsiveness.

**Conclusion:**

This review highlights shared neural abnormalities (Triple Network model) and introduces thalamocortical dysrhythmia as a novel explanatory framework linking FND and CP. Future research should target populations with coexisting disorders, potentially paving the way for innovative treatments, including hypnosis and neuromodulation/neurofeedback.

**WHAT IS ALREADY KNOWN ON THIS TOPIC**

- Functional neurological disorder (FND) and chronic pain frequently co-occur and share overlapping clinical features. Although both conditions have been studied individually, little is known about their shared neurobiological mechanisms.

**WHAT THIS STUDY ADDS**

- This review identifies consistent evidence of shared dysfunction in brain networks related to sensorimotor control, emotional regulation and self-referential processing across FND and chronic pain. It also highlights thalamocortical dysrhythmia as a potential unifying mechanism.

**HOW THIS STUDY MIGHT AFFECT RESEARCH, PRACTICE OR POLICY**

- Understanding shared neural mechanisms may inform the development of unified therapeutic strategies such as neuromodulation or neurofeedback/hypnosis. It also underscores the need for integrated clinical approaches and future studies targeting patients with both FND and chronic pain.

## Introduction

Functional neurological disorder (FND) is a complex, common and disabling neurological condition characterised by neurological symptoms and signs without objective findings on diagnostic tests.^1 2^ Chronic pain (CP), defined as pain that persists for more than 12 weeks despite treatment,^3^ is one of the most frequently reported comorbidities in patients with FND. Their symptoms frequently interact within a complex, self-sustaining cycle, complicating treatment efforts, as each condition may serve simultaneously as a precipitating factor and a perpetuating influence for the other.^4^,^8^ A recent systematic review and meta-analysis reported pain in approximately 55% of FND patients, notably higher among those with functional movement disorders (61%) and functional seizures (FS, 42%). CP often precedes and predicts poorer outcomes in FND.^9^

The relationship between pain and FND dates back to Gowers, who associated pain with ‘hysteria’.^10^ Modern studies reinforce this link; fibromyalgia (FM) predicts FS diagnosis with a 75% positive predictive value,^4 11^ and FS accounts for 75% of paroxysmal events in FM patients, compared with 11% for epilepsy.^12^ In another study on people with FS,^13^ 76% of patients reported moderate-to-severe pain of any type, showing a higher-than-usual frequency of pain symptoms, as compared with the general European population (18%).^14^ These robust clinical and epidemiological associations between FND and CP suggest a potential convergent neurobiological mechanism for both conditions, as both FND and CP are linked to psychological factors like trauma and stress.^15^,^19^

Understanding their shared mechanisms could improve treatments,^20 21^ and common biomarkers could unveil novel therapeutic targets.^22 23^

## Methods

We reviewed neurophysiological and functional neuroimaging studies in patients with FND and CP and used the following databases: Medline/PubMed, SpringerLink, Science Direct, Ovid, Scopus, CInAHL/EBSCO and Cochrane Library. The search syntaxes were created using keywords and MeSH terms related to FND and CP, as well as neurophysiological and neuroimaging methods. The search syntaxes were agreed on by all authors, and the search included studies conducted from 1990 to December 2024. The searches were conducted initially by KP (2021) and updated in 2024 by SK. A full list of the search terms used is reported in online supplemental table 1.

Studies were included if they:

- Involved patients with a diagnosis of FND or CP.
- Aimed to investigate the neurobiological basis of FND or CP.
- Used electroencephalogram (EEG), magneto-encephalography (MEG), functional MRI (fMRI), positron emission tomography (PET) or single photon emission computed tomography (SPECT).
- Were published in English.

The following data were extracted from each study: the first author’s last name, publication year, sample size, study design, type of FND and CP, and type of neuroimaging used. Data were extracted by one reviewer (SK) and checked for accuracy by a second independent reviewer (AD). Disagreements were resolved by discussion.

**Figure 1:**
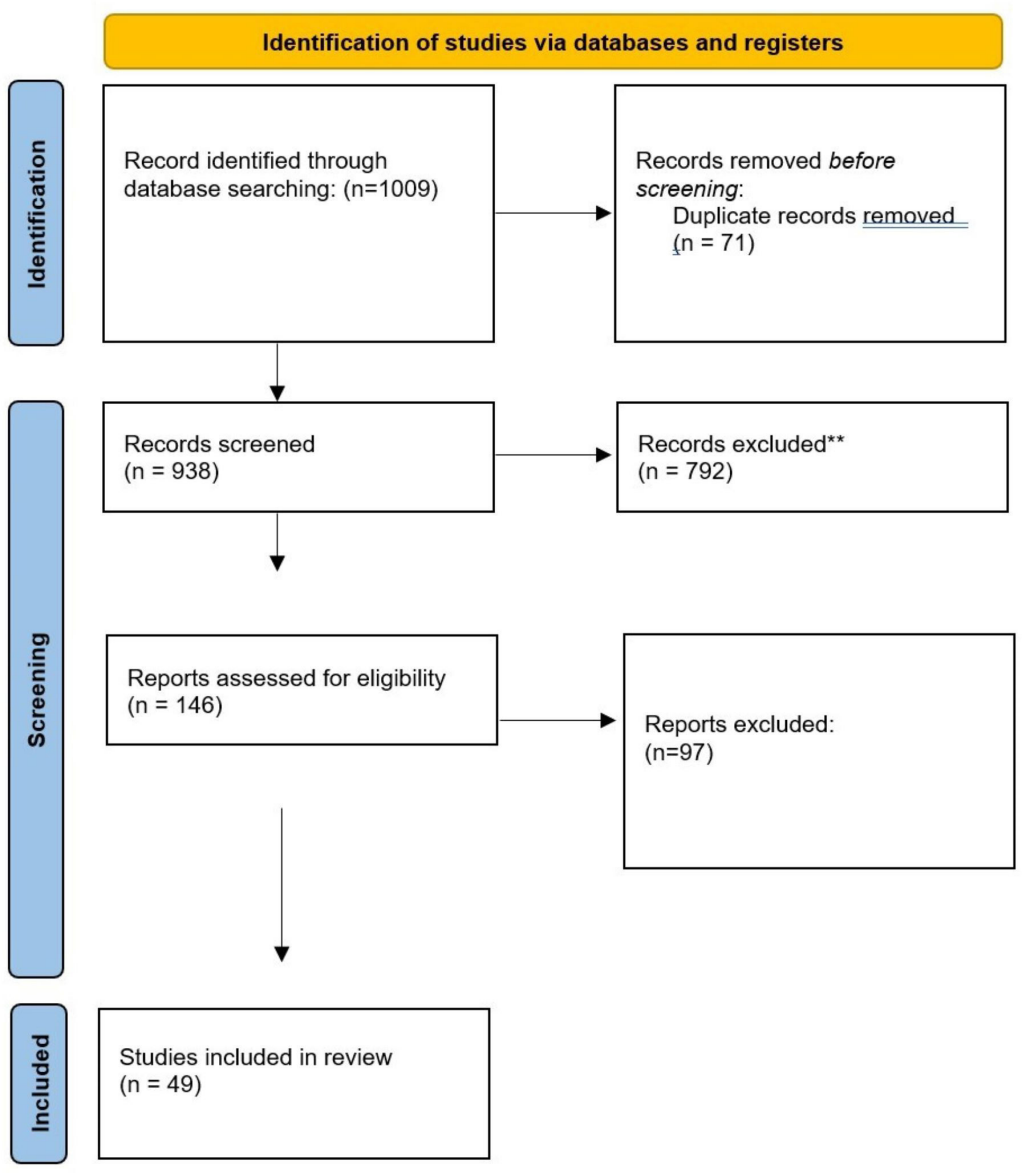
Studies included for functional neurological disorder.

Relevant literature cited within the publications identified was manually retrieved. Single case reports, commentaries, editorials, non-peer-reviewed publications and grey literature were not included. A further search on the search engine Google Scholar was also performed to identify potential studies that could be added to the results obtained from the database searches (figures1  2).

**Figure 2:**
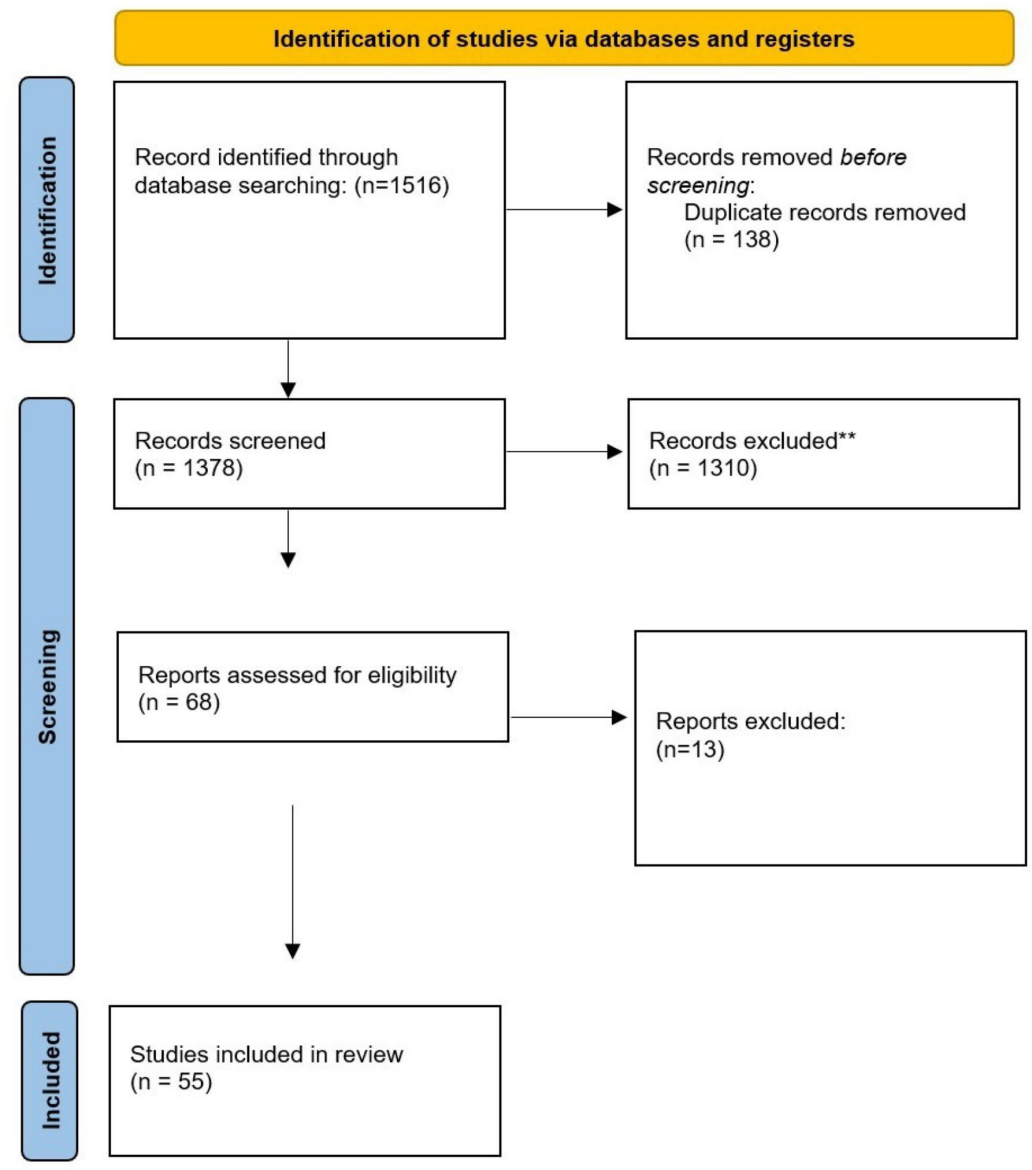
Studies included for chronic pain.

Due to the heterogeneous nature of the data, results were analysed thematically and presented as a narrative synthesis. Quantitative methods (eg, funnel plots, Egger’s test) for assessing publication bias were infeasible, as pooled effect sizes and standard errors were unavailable. Formal quality appraisal (eg, GRADE, Newcastle-Ottawa Scale) was also omitted due to the exploratory nature. However, detailed summary tables (online supplemental tables 2,3) outlining samples, imaging methods and key findings are provided to ensure transparency.

## Results

The results are organised by electrophysiological and neuroimaging modality, and each subsection highlights key findings from EEG, MEG, fMRI, PET and SPECT studies. online supplemental material

### Electroencephalogram (EEG) studies

#### Electroencephalogram (EEG) correlates of functional neurological disorder (FND)

1. Beta frequency changes

- Increased beta: A quantitative EEG (QEEG) study in FS patients reported higher 13–30 Hz beta activity over left central sites (C3) compared with controls, suggestive of cortical overactivation of fronto-parietal and sensorimotor cortices.^24^
- Pre-attack beta suppression: Another QEEG investigation found a decrease in beta power at central electrodes (C3, C4, Cz) preceding FS attacks, an effect distinct from epileptic seizures.^25^ This is reminiscent of event-related desynchronisation prior to voluntary movement, possibly reflecting a maladaptive anticipation of motor activity modulated by dopaminergic pathways.^26^

1. Gamma frequency changes

- Increased gamma in left parietal regions suggests heightened sensorimotor processing in FS.^24^
- Reduced gamma in the right superior temporal gyrus^27^ or between frontal and posterior regions^28^ may reflect aberrant emotional processing in FS (eg, regulating stress responses).

1. Alpha band connectivity

- Altered alpha connectivity involving basal ganglia, limbic regions, prefrontal, temporal, parietal and occipital cortices has been reported in FS compared with healthy controls.^29^ Graph-theoretic measures show reduced small-worldness that correlates with monthly FS frequency, reflecting global network dysregulation.^30^

1. Differentiation from epileptic seizures

- Certain EEG-based features (eg, limited dominant frequency variation on Fast Fourier Transforms and rhythmic artefacts) help distinguish ‘convulsive’ FS from epileptic seizures.^31^

1. Functional motor disorders (FMD)

- Patients with FMD show reduced inferior parietal cortex (IPC) modulations (C-cluster) and altered inferior frontal gyrus (IFG) modulations (R-cluster), further implicating frontoparietal and motor networks.^32^

#### Electroencephalogram (EEG) correlates of chronic pain (CP)

1. Alpha power changes

- Patients with spinal cord injury and CP demonstrate decreased alpha power over frontal regions.^33 34^ Acute pain studies^35^ and capsaicin-induced muscle pain also show alpha power reduction.^36^
- Attention to pain further suppresses alpha activity^37^ which may increase cortical excitability via a ‘thalamocortical gate’ mechanism.^38 39^

1. Beta frequency changes

- High beta activity from frontal, central and parietal regions is significantly associated with self-reported pain intensity in chronic low back pain.^40^
- Psychological interventions (eg, mindfulness) may reduce beta power in cortical regions, correlating with decreased pain intensity.^41^

1. Theta and delta power

- Increased theta and delta seen in CP conditions such as migraine and chronic pelvic pain,^42^,^44^ and FM.^45 46^ Elevated theta in prefrontal and anterior cingulate cortices (ACC) may reflect persistent emotional or cognitive distress.

1. Thalamocortical dysrhythmia (TCD)

- Abnormal interactions between theta- and beta-generating regions can produce the so-called ‘edge effect’, where a central theta-hypoactive region is surrounded by beta-hyperactive areas. This pattern is observed in multiple CP syndromes, including FM, supporting the idea of widespread network-level maladaptive oscillations^47 48^

#### Overlap in functional neurological disorder (FND) and chronic pain (CP) (electroencephalogram (EEG))

Both conditions show overactivation of sensorimotor and fronto-parietal regions (beta/gamma changes) alongside impaired inhibitory or emotional regulatory mechanisms (altered alpha/gamma). Increased theta and disrupted thalamocortical rhythms also appear in both, indicating a shared pathophysiology involving excessive cortical excitability and ineffective gating of sensory or emotional signals.

### Magneto-encephalography (MEG) studies

#### Magneto-encephalography (MEG) studies in functional neurological disorder (FND)

1. Reduced occipital alpha and increased low-frequency power in fronto-temporal regions suggest enhanced fronto-limbic excitability.^49^
2. Emotional processing tasks implicate an unchanged automatic emotional salience detection but with abnormal engagement of sensorimotor and posterior networks in patients with functional weakness and/or sensory disturbance.^50^

#### Magneto-encephalography (MEG) studies in chronic pain (CP)

1. High alpha power ratio (low:high) across multiple cortical regions in CP.^51^
2. Increased theta power in the DMN (default mode network) and decreased gamma in DMN/ascending nociceptive pathway are emerging potential ‘signatures’ of CP.^52^
3. Pain relief interventions (eg, deep brain stimulation) show reduced ACC activation, supporting the ACC’s crucial role in affective pain processing.^53^
4. FM research using MEG reveals:

- Reduced DMN–insula connectivity at theta band and negative correlation with the number of tender points at beta band.^54^
- Increased theta in prefrontal/orbitofrontal cortex, excess beta/gamma in insular and sensorimotor cortices.^55^

1. Complex regional pain syndrome correlates with reduced somatosensory and precuneus activity, pointing to DMN involvement in abnormal pain perception.^56^
2. Temporomandibular disorder and central post-stroke pain also show prolonged cortical dipole activation and beta/gamma augmentation in parietal/frontal cortices.^57 58^

#### Overlap in functional neurological disorder (FND) and chronic pain (CP) (magneto-encephalography (MEG))

Both FND and CP populations show elevated low-frequency (theta/delta) activity in fronto-limbic and DMN regions, alongside disrupted sensorimotor integration. This dysregulation reflects a breakdown in the coordination between emotional salience processing (via limbic structures), self-referential networks (DMN) and motor control systems—suggesting a shared core mechanism that might be related to symptom generation/persistence in both conditions.

### Functional MRI (fMRI) studies

#### Functional MRI (fMRI) studies in functional neurological disorder (FND)

1. Resting state networks

- Increased connectivity among fronto-parietal, sensorimotor, executive and DMN.^59^,^62^ Greater connectivity correlates with higher FS frequency, suggesting that excess crosstalk between emotional, executive and motor areas predisposes to dissociative attacks.

1. Involvement of limbic structures

- Heightened amygdala and insula interactions with motor regions; hyperconnectivity in the right amygdala–IFG axis in FS when compared with healthy controls.^63^ These patterns reflect emotional dysregulation bleeding into motor control circuits. A mixed group of FND patients (FS, FMD, PPPD) showed insular co-(de)activation patterns compared with the salience network (SN), the somatomotor network and the DMN, compared with the controls.^64^ Moreover, in FND subjects, these dynamic alterations conjointly correlated with salivary amylase measures (marker of stress) and duration of symptoms.^64^ Additionally, increased amygdala activity was noted during cognitive reappraisal compared with controls in a predominantly FMD cohort.^65^

1. Effective connectivity

- Studies applying EC show inhibitory influences from limbic (eg, amygdala, ACC) onto motor/executive areas (eg, insula, IFG) in FS, disrupting normal volitional motor control in FS.^66^,^69^

1. Delay in diagnosis

- Individuals with delayed FS diagnosis can have unique connectivity patterns, such as greater bilateral posterior cingulate cortex (PCC) and left anterior insula activation to stress.^70 71^

1. Structural-functional coupling

- FS patients exhibit more lattice-like networks and reduced structural–functional connectivity, particularly within attention, sensorimotor, subcortical and DMN regions.^72^,^75^ In mixed FND populations, increased functional connectivity between motor and insular regions correlated with symptom severity and clinical improvement,^76^ while significant shifts in Temporo-Parietal Junction (TPJ) and precuneus centrality were seen in functional weakness in FMD.^77^ Wegrzyk *et al* found the caudate and amygdala hyperconnectivity in FND,^78^ whereas diminished functional connectivity from sensorimotor cortices to key areas (such as the SMA, insula, and dorsal ACC) was described in FMD.^79 80^ Sojka *et al* demonstrated heightened activation of sensorimotor and associative cortices (precuneus, post-central gyrus, PCC) in FND patients viewing negative stimuli,^81^ and Baek *et al* noted reduced inferior parietal engagement during involuntary versus voluntary movement, aligning with disrupted intention.^82^ Bühler *et al*^83^ further underlined the role of the TPJ in agency misjudgments.^83^
- These findings highlight maladaptive limbic–motor interactions, particularly disrupted connectivity in emotional processing, sensorimotor integration and self-agency networks. The amygdala, TPJ, and precuneus consistently emerge as key nodes linking emotional salience to motor execution. Persistent amygdala hyperactivity correlates with abnormal sensorimotor responses, underscoring deficits in top-down control and agency within limbic–motor networks central to FND pathophysiology.^84 85^ Overall, impaired top-down regulation and self-agency are fundamental to FND neurobiology.
- Therapeutically, multidisciplinary motor retraining reduced motor symptoms and modified amygdala connectivity. Positive outcomes corresponded with enhanced amygdala–ventromedial prefrontal connectivity, whereas poorer responses correlated with stronger amygdala–primary motor cortex connectivity,^86^ demonstrating that targeted interventions recalibrating emotional influence toward prefrontal executive networks may be important in treating FND.

#### Functional MRI (fMRI) studies in chronic pain (CP)

1. Acute versus chronic

- While acute pain typically activates sensorimotor cortex, insula and ACC, CP shows stronger involvement of prefrontal cortex (linked to the DMN).^87 88^

1. Connectivity alterations

- Increased insula–DMN connectivity in FM.^89^,^93^
- Greater ACC–basal ganglia–sensorimotor connectivity also noted, correlating with pain intensity.^94^,^100^
- In CLBP, heightened medial prefrontal to ACC/insula connectivity suggests persistent salience/emotional modulation.^101^,^103^ Equivalent results were found in comparable studies that investigated the fMRI changes in patients with chronic back pain.^104^,^107^

1. Stimulation-induced state changes

- Pain stimulation can further increase connectivity among thalamus, insula, precuneus and other DMN nodes in CP patients.^108^,^110^
- Deep brain stimulation of the ventral striatum/anterior limb of the internal capsule (vs/ALIC) leads to reduced orbitofrontal cortex activation, highlighting a modulatory effect on affective pain circuits^111 112^

1. Complex regional pain syndrome

- Marked by excess thalamo-somatosensory connectivity, with additional involvement of the IFG and orbitofrontal cortex.^113 114^

#### Overlap in functional neurological disorder (FND) and chronic pain (CP) functional MRI (fMRI)

Shared alterations in ACC, insula, sensorimotor areas and DMN. Increased connectivity between emotional processing centres (eg, amygdala, insula) and motor/executive networks is common. Dysregulated self-referential processing (DMN) and salience/emotion processing (limbic regions) underlie key symptoms in both conditions.

### Positron emission tomography (PET) findings

#### Positron emission tomography (PET) studies in functional neurological disorder (FND)

- Hypometabolism in right IPC and bilateral ACC is seen in FS, correlating with cerebellum and left hippocampal gyrus dysfunction.^115 116^
- Hypermetabolism in the right PCC (involved in the DMN) in conversion disorder, suggesting excess self-referential or arousal processing.

#### Positron emission tomography (PET) studies in chronic pain (CP)

- Increased uptake in cingulate cortex, thalamus and amygdala in chronic arthritic knee pain versus acute experimental knee pain.^117^
- CP from brachial plexus avulsion: decreases in right thalamus metabolism alongside increases in orbitofrontal cortex, insula and DLPFC, implicating affective and evaluative components of CP.^118^,^121^
- Additionally, a significant body of PET studies in CP underscore the critical involvement of opioid and dopaminergic systems in pain’s affective and motivational components.^122^ In FM, PET imaging demonstrates reduced mu-opioid receptor (MOR) binding potential (BP) in key pain-modulating regions, including the nucleus accumbens, amygdala, and dorsal cingulate cortex. Lower MOR BP in the nucleus accumbens correlates with higher affective pain ratings, with similar negative correlations observed throughout the cingulate cortex and striatum.^123^

- Patients with chronic non-neuropathic back pain (CNBP) exhibit reduced dopamine D2/D3 receptor availability in the ventral striatum, linked to positive affect, pain tolerance and affective pain dimensions. During acute pain challenges, CNBP patients show decreased dopamine release compared with controls, correlating with altered endogenous opioid activity in the amygdala. Collectively, these findings implicate dopaminergic and opioid systems in CP’s motivational and affective components.^124^
- In burning mouth syndrome, significantly decreased presynaptic dopaminergic function is observed in the putamen, indicated by reduced Fluorodopa (18F) (FDOPA) uptake. This aligns with prior neurophysiological evidence of impaired dopaminergic inhibition, directly implicating nigrostriatal dopaminergic dysfunction in this CP disorder.^125^

#### Overlap in functional neurological disorder (FND) and chronic pain (CP)

Both demonstrate significant cingulate and parietal involvement. Thalamic and orbitofrontal/limbic metabolic changes appear across FS and various CP states, reiterating a common subcortical–cortical dysregulation underpinning emotional, attentional, and sensory processing.

### Single photon emission computed tomography (SPECT) findings

#### Single photon emission computed tomography (SPECT) studies in functional neurological disorder (FND)

1. Perfusion changes

- During FS, decreased perfusion has been noted in the posterior parietal cortex.^126^ ^127^ ^117^ ^128^
- Some FS patients show increased perfusion in areas overlapping the DMN (eg, right precuneus and right PCC),^129^ hinting at DMN overactivity during episodes.^130^
- When comparing psychogenic tremor vs essential tremor, a study in FMD showed increased Regional cerebral blood flow (rCBF) in left IFG and insula, with reduced rCBF in anterior DMN regions during motor tasks.^131^

1. Subcortical structures

- SPECT findings in conversion disorder highlight rCBF reductions in the thalamus and basal ganglia contralateral to functional deficits, which normalise on symptom resolution.^132^

#### Single photon emission computed tomography (SPECT) studies in chronic pain (CP)

- Reduced prefrontal cortex and thalamic perfusion in chronic back pain.^133^,^136^
- Increased thalamic and cingulate perfusion in somatoform pain, but decreased in frontal, occipital and left temporal regions.^137^,^139^
- FM studies show decreased perfusion in frontal, temporoparietal, right precuneus and right PCC—paralleling certain FS findings.^140^,^142^

#### Overlap in functional neurological disorder (FND) and chronic pain (CP)

Similar patterns of DMN region hyper- or hypoperfusion (PCC, precuneus) and thalamic involvement emerge in both. The interplay of limbic–subcortical circuits (eg, thalamus and basal ganglia) and cortical networks (frontal/parietal lobes) again demonstrates a shared pathophysiological substrate.

## Discussion

Our review synthesises neurophysiological and neuroimaging findings across FND and CP, focusing on shared mechanisms rather than effect sizes. Although the included studies vary in quality and design, we identified recurring patterns—across modalities and patient groups—that support a convergent neurobiological model. These patterns are not drawn from isolated reports but reflect thematic overlaps identified in multiple independent studies, summarised in online supplemental tables 2 and 3. These patterns point towards shared network dysfunction in emotion processing, sensorimotor control and default mode activity. These similarities may help explain why FND often coexists with CP conditions and highlight potential shared targets for therapeutic intervention.

Below, we synthesise these findings.

### Overactivation of sensorimotor networks

Both FND and CP patients exhibit overactivation in sensorimotor regions.^24 90^ In FND, this may stem from maladaptive emotional responses that amplify motor excitability, whereas in CP, persistent nociceptive input may sensitise these same pathways. Functional MRI studies in CP show increased medial prefrontal cortex activity and connectivity with the anterior cingulate cortex, secondary somatosensory cortex and insula.^101^ By extension, excessive sensorimotor or limbic activation during pain could occasionally ‘spill over’, overpowering executive control and precipitating FND events—consistent with evidence of heightened limbic-sensorimotor excitability in FND.^49^ Hence, both conditions illustrate an intricate interplay among stress, pain and movement circuits.

### Alterations in the default mode network (DMN) and functional connectivity

Dysfunction within the DMN, a network central to self-referential processing, is consistently reported in both FND and CP. In FND, abnormal DMN hyperconnectivity may predispose patients to dissociative-like states in response to external stressors. In CP, altered DMN function could underlie sustained pain perception even in the absence of ongoing nociceptive stimuli.^143^ Notably, both FND and FM patients show decreased perfusion in the right precuneus and right posterior cingulate gyrus,^129 144^ regions commonly linked to the DMN. Additionally, FND exhibits stronger connectivity between emotion-processing and sensory integration areas, potentially impairing movement execution and self-perception.^145^ In CP, similar alterations in functional connectivity (eg, between frontolimbic and sensorimotor circuits) help explain the persistence of pain despite the resolution of the initial peripheral cause.^49 53 61^

### Shared emotional processing Errors

Common disturbances in emotional regulation networks (anterior cingulate cortex, insula and amygdala) underscore the role of affective factors in both FND and CP. These regions are central to the SN, which detect and prioritise emotionally significant stimuli. Emotional triggers can provoke/exacerbate FND (eg, heightened arousal, stress)^64 73 74^ or exacerbate CP.^126^ Thus, the SN over-responsiveness represents a potential unifying mechanism.

#### Thalamocortical dysrhythmia (TCD) and its impact on movement and perception

Thalamocortical dysrhythmia (TCD) provides a compelling framework for both conditions.^44 48^ In TCD, reduced thalamic drive can induce low-frequency theta rhythms surrounded by compensatory beta overactivation (the ‘edge effect’). In CP, TCD maintains sustained nociceptive hypersensitivity; in FS, overactive beta oscillations, influenced by dopaminergic pathways, may predispose individuals to seizure-like motor discharges.^26^ By extension, TCD can also affect DMN regulation, because the thalamus is essential for filtering information before it reaches higher cortical areas. Disruption here might exacerbate maladaptive self-focus on pain or distress, thus linking TCD to both CP experiences and functional seizures.

### Shared role of alpha Oscillations

Both conditions reveal characteristic changes in alpha power—often interpreted as an index of inhibitory gating and cortical ‘idling’. FS patients show reduced occipital alpha power,^49^ while CP patients frequently present with decreased frontal alpha power.^30^ Such alpha suppression has been linked to heightened attention to internal or external stimuli; thus, in FS, an emotional or interoceptive trigger could promote excessive alpha suppression, facilitating a seizure-like event. Similarly, in CP, diminished alpha might enhance cortical responsiveness to nociceptive input and amplify pain perception.

The integrative cognitive model of FS posits that functional seizures arise partly from defective inhibitory mechanisms.^146^ Emotional overload or stress may activate a ‘seizure scaffold’, wherein maladaptive sensorimotor and limbic integration produces FS. Clinically, CP could function as another potent stressor, triggering FS in susceptible individuals. Detailed characterisation of pain in FND populations—and vice versa—is therefore vital to clarify how each might perpetuate the other.

### Triple network model

Involvement of three core networks—salience network (SN), DMN and central executive network (CEN)—can be framed within the triple network model, a foundational framework for understanding neural dysregulation in neuropsychiatric disorders.^147 148^ These networks interact dynamically to regulate sensory, emotional and cognitive processes. The SN (anterior insula, anterior cingulate cortex) identifies salient stimuli (eg, pain or internal disturbances) as significant, prompting the DMN (involved in self-referential processing) to integrate these sensations into an internal narrative, potentially amplifying subjective distress. Concurrently, maladaptive engagement or insufficient regulation by the CEN (involved in goal-directed behaviour) may foster ineffective coping or heightened rumination. Hence, in FND and CP, this dysfunctional interplay may be the source of symptom generation and persistence (eg, seizures, abnormal movements, CP). Although the triple network model is hypothesis-generating, its strength lies in consistent multimodal evidence (EEG, MEG, fMRI, PET, SPECT) and shared oscillatory dysregulation (alpha/beta), reinforcing the concept of a common neurobiological substrate.

However, similar SN–DMN–CEN disruptions are also seen in depression, anxiety and Post-traumatic stress disorder (PTSD), frequently comorbid with FND/CP.^147 149^ Future research using psychiatric control groups and matched symptom designs is crucial to clarify if these network alterations represent core FND/CP features or broader transdiagnostic processes such as heightened salience attribution, impaired emotional regulation or altered self-referential processing.^150^

### Cue for novel therapeutic approaches

From a therapeutic standpoint, neuromodulation and neurofeedback approaches targeting aberrant beta and alpha power have shown promising results in CP, with up to 82% pain reduction reported in some studies.^151^ Similar approaches may hold promise for FND management, given that both conditions exhibit overlapping rhythmic and network dysregulations.^152 153^

Another important tool would be the use of hypnosis, which has been proven effective in both FND^154^ and CP.^155 156^ This can be explained by the fact that hypnosis often shows the opposite changes in DMN and SN, as seen in FND and CP.^157^

Looking forward, clarifying the neurobiological links between FND and CP will inform the design of advanced therapeutic strategies—potentially addressing shared dysrhythmias across emotion, cognition and sensorimotor domains.

### Limitations

This review has several limitations. First, most neuroimaging research in FND focuses on FS, potentially limiting generalisability. Second, we did not formally assess study quality or bias, constraining the interpretation of evidence. Our narrative synthesis highlights common neurobiological mechanisms across various methods and populations, but the lack of standardised quality ratings and pooled analyses restricts inferential depth. Third, limiting searches to English-language, peer-reviewed studies and excluding grey literature may introduce bias. Fourth, many included studies were cross-sectional and underpowered, weakening conclusions. Finally, the lack of standardised outcome measures and heterogeneity in imaging protocols prevented statistical synthesis, emphasising the need for future multimodal, longitudinal research with standardised methods.

Additionally, many studies did not control for psychiatric comorbidities such as depression and anxiety, prevalent in both FND and CP. Given overlapping network-level alterations across these conditions, as noted by Davis *et al*,^150^ it remains unclear if reported neural signatures are specific to FND or CP or reflect broader transdiagnostic processes, thus limiting interpretation as disorder-specific biomarkers.

## Conclusions

To our knowledge, this is the first review synthesising neuroimaging and neurophysiological findings in both FND and CP—conditions that frequently overlap and co-occur clinically. The evidence highlights shared maladaptive neural responses to emotional and nociceptive stressors, with consistent disruptions across salience, sensorimotor and self-referential networks. Clarifying the specificity of these neural signatures will critically enhance the development of precise, mechanism-based interventions.

## Supplementary material

10.1136/bmjno-2025-001032 online supplemental file 1

## Data Availability

Data sharing not applicable as no datasets generated and/or analysed for this study.
