# Supplementary material for: Shared neural signatures in Functional Neurological Disorder and Chronic Pain: a multimodal narrative review
Source: BMJ Neurol Open. 2025 Jul 13;7(2):e001032. doi: 10.1136/bmjno-2025-001032 (PMC12258340; doi:10.1136/bmjno-2025-001032)
Supplement: online supplemental file 1 [file bmjno-7-2-s001.docx]

Supplementary Material:

**Table 1: Search Terms**

The searches were conducted using the following terms:

| (“Functional neurological disorder” OR “FND” OR “functional movement disorder” “functional motor disorder” OR “Conversion Disorder” OR "Nonepileptic Attack Disorder" OR "Psychogenic Nonepileptic Seizure " OR "Functional Seizures" OR "dissociative seizures" OR “NEAD” OR “PNES” OR “pseudoseizure”) | **AND** | ("fMRI" OR "functional MRI" OR "functional magnetic resonance imaging" OR “PET” OR “Positron-Emission Tomography” OR “Positron Emission Tomography”) OR “MEG” OR “Magnetoencephalography”) OR “SPECT” OR “Single-photon emission computed tomography”) |
| --- | --- | --- |
| Chronic Pain - (“Fibromyalgia” OR “Chronic Pain” OR “Persistent pain” OR “Chronic generalized pain” OR “Chronic widespread pain” | **AND** | ("fMRI" OR "functional MRI" OR "functional magnetic resonance imaging" OR “PET” OR “Positron-Emission Tomography” OR “Positron Emission Tomography”) OR “MEG” OR “Magnetoencephalography”) OR “SPECT” OR “Single-photon emission computed tomography”) |

**Table 2: Studies in FND**

| Study | Sample size | Patients included – Type | Study design | Methodology | Results | References | |
| --- | --- | --- | --- | --- | --- | --- | --- |
| **EEG** | | | | | | | |
| Arikan et al 2021  10.1177/1550059420918756 | 39 | psychogenic non-epileptic seizures (PNES) | Cohort | EEG | Six separate EEG band power, namely (C3-high beta, C3-gamma, C3-gamma-1, C3-gamma-2, P3-gamma, P3 gamma-1), were found to be higher in the patients diagnosed with PNES than in the control group. Our findings show that PNES correlate with high-frequency oscillations on central motor and somatosensory cortices | [25] | |
| Barzegaran et al 2016  10.1136/jnnp-2014-309483 | 18 | PNES | Cohort | EEG | The significant effect of PNES-a decrease in lagged Functional connectivity between the basal ganglia and limbic, prefrontal, temporal, parietal and occipital regions-was found in the α band. | [30] | |
| Umesh et al 2017  10.1016/j.psychres.2016.11.003 | 15 | PNES | Cohort | EEG | Statistically significant gamma spectral power, cortical sources and connectivity pattern was found in some brain areas. Region specific aberrant gamma activity and its relationship to psychopathology are discussed. | [28] | |
| Vinton et al 2004  10.1111/j.0013-9580.2004.04704.x | 15 | PNES | Cohort | EEG | The dominant frequency remained stable within a narrow range for the duration of the PNES, whereas in the epileptic seizures, it evolved through a wide range. The coefficient of variation of the frequency during the seizures was considerably less for patients without epilepsy (median, 15.0%; range, 7.2-23.7% vs. median, 58.0%; range, 34.8-92.1%; p < 0.001). The median frequency did not differ significantly between groups (4.2 vs. 4.6 Hz; p = 0.290). | [32] | |
| Xue et al 2013  10.1177/0300060513496170 | 15 | PNES | Cohort | EEG | Difference topology revealed that patients with PNES had decreased long linkage between the frontal region and other regions compared with controls. There were no significant between-group differences in global efficiency. Neuropsychological scores were significantly higher in patients than controls, but there were no correlations with network properties. | [29] | |
| Weissbach et al 2023  10.1002/mds.29458 | 21 | FMD | Cohort | EEG | Temporal decomposition allowed to distinguish between EEG codes reflecting sensory (S-cluster), motor (R-cluster), and integrated sensory-motor processing (C-cluster). Our study shows that FMD is characterized by altered integration of sensory information with motor processes | [33] | |
| **fMRI** | | | | | | |  |
| Aybek et al 2015  10.1371/journal.pone.0123273 | 12 | Conversion disorder | Cohort | fMRI | We found increased amygdala activation to negative emotions in CD compared to healthy controls in region of interest analyses, which persisted over time consistent with previous findings using emotional paradigms. Furthermore during whole brain analyses we found significantly increased activation in CD patients in areas involved in the 'freeze response' to fear (periaqueductal grey matter), and areas involved in self-awareness and motor control (cingulate gyrus and supplementary motor area). | [85] | |
| Allendorfer et al 2019  10.1016/j.nicl.2019.101967 | 12 | psychogenic non-epileptic seizures (PNES) | Cohort | fMRI | There was stress fMRI hyporeactivity in left/right amygdala and left hippocampus in PNES versus HCs (corrected p < .05). PNES exhibited a positive associationDieetween alpha-amylase change and right amygdala activation (rs = 0.71, p = .010). PNES versus HCs exhibited greater right amygdala rs-FC to left precentral and inferior/middle frontal gyri (corrected p < .05). | [64] | |
| Amiri et al 2021  10.1016/j.yebeh.2021.108085 | 23 | psychogenic non-epileptic seizures (PNES) | Cohort | fMRI | Compared to the HC subjects, in patients with PNES: (i) the left insula (INS) and left and right inferior frontal gyri (IFG) are more inhibited by the amygdala (AMYG), anterior cingulate cortex (ACC), and precentral gyrus (PCG); (ii) the left AMYG has greater inhibitory effects on the INS, IFG, dorsolateral prefrontal cortex (DLPFC), PCG, and supplementary motor area (SMA); (iii) the left ACC has more inhibitory effects on the INS and IFG; (iv) the right ACC is more inhibited by the INS and IFG, and has a less inhibitory effect on the SMA and PCG; and (v) the left caudate (CAU) had increased inhibitory effects on the AMYG and IFG and a more excitatory effect on the SMA. | [68] | |
| Baek et al 2017  10.1017/S0033291717000071 | 26 | FND | Cohort | fMRI | The results converge with observations of low inferior parietal activity comparing involuntary with voluntary movement in FND, emphasizing core deficiencies in intention. Heightened precision of this impaired intention is consistent with Bayesian theories of impaired top-down priors that might influence the sense of involuntariness. | [83] | |
| Blakemore et al 2016  10.1016/j.neuropsychologia.2016.11.005 | 10 | Conversion disorder | Cohort | fMRI | controls showed increased activity in the inferior frontal cortex and pre-supplementary motor area, whereas patients had greater activity in the cerebellum (vermis), posterior cingulate cortex, and hippocampus. Engagement of a cerebellar-limbic network in patients is consistent with heightened processing of emotional salience, and supports the role of the cerebellum in freezing responses in the presence of aversive events. | [88] | |
| Bühler et al 2024  10.1136/bmjno-2023-000525 | 23 | FND | Cohort | fMRI | At baseline, FND patients showed decreased accuracy in detecting reduced agency compared with controls (p<0.001), paralleled by lower brain activation in the rTPJ during MA (p=0.037, volume of interest). | [84] | |
| Burke et al 2014  10.1016/j.nicl.2014.09.016 | 10 | Conversion disorder | Cohort | fMRI | Group analyses revealed 10 areas that had significantly greater activation (p < .05) when stimulation was applied to the anesthetic body part compared to the contralateral sensate mirror region. They included right paralimbic cortices (anterior cingulate cortex and insula), right temporoparietal junction (angular gyrus and inferior parietal lobule), bilateral dorsolateral prefrontal cortex (middle frontal gyri), right orbital frontal cortex (superior frontal gyrus), right caudate, right ventral-anterior thalamus and left angular gyrus. | [65] | |
| Diez et al 2019  10.1136/jnnp-2018-319657 | 30 | FND | Cohort | fMRI | Compared to controls, patients with FND exhibited increased SFC from motor regions to bilateral posterior insula, temporoparietal junction (TPJ), middle cingulate cortex, and putamen.  Symptom severity correlated with increased SFC from the left anterior insula to right anterior insula and TPJ, with clinical improvement correlating with increased SFC from the left centromedial amygdala to right anterior insula. | [77] | |
| Ding et al 2013  10.1371/journal.pone.0063850 | 17 | psychogenic non-epileptic seizures (PNES) | Cohort | fMRI | Many regional characteristics were altered in structural connectivity network, involving attention, sensorimotor, subcortical and default-mode networks. These regions with altered nodal characteristics likely reflect disease-specific pathophysiology in PNES. | [73] | |
| Ding et al 2014  10.1016/j.eplepsyres.2014.05.006 | 18 | PNES | Cohort | fMRI | We found that patients with PNES showed abnormal FCD regions mainly in the frontal cortex, sensorimotor cortex, cingulate gyrus, insula and occipital cortex. Seed-voxel correlation analyses also showed disrupted functional connectivity between these regions. In addition, the occipital cortex FCD correlated with duration of disease. | [74] | |
| Faul et al 2020  10.1016/j.pscychresns.2020.111125 | 14 | FMD | Cohort | fMRI | Baseline severity was associated with greater primary motor cortex (M1) activation, and greater improvement correlated with more significant changes in M1 activation. | [87] | |
| Goodman et al 2022  10.1111/epi.17179 | 49 | Functional seizures | Cohort | fMRI | Responses to stressful math performance within the left anterior insula and functional connectivity between the anterior insula seed region and a precentral gyrus cluster were significantly negatively correlated with time lag to diagnosis for the early but not the delayed FS diagnosis group. There was no correlation between fMRI findings and psychiatric symptoms. | [63] | |
| Goodman et al 2022  10.1136/jnnp-2022-329838 | 52 | Functional seizures & Traumatic Brain injury | Cohort | fMRI | Five linear mixed-effects analyses identified clusters of connectivity coefficients that differed between groups within the posterior cingulate of the default mode network, insula and supramarginal gyrus of the executive control network and bilateral anterior cingulate of the salience network (all α=0.05, corrected). Cluster signal extractions revealed decreased contributions to each network for FS+TBI compared to TBI only. | [72] | |
| Hassa et al 2017  10.1016/j.nicl.2017.04.004 | 13 | Conversion disorder | Cohort | fMRI | increased functional connectivity in patients between the left amygdala and the (pre-)supplemental motor area and the subthalamic nucleus, key regions within the motor control network. These findings suggest a novel mechanistic direct link between dysregulated emotion processing and motor control circuitry in conversion disorder. | [86] | |
| Hassa et al 2021  10.3389/fpsyt.2021.613156 | 24 | FND | Cohort | fMRI | Patients with FND showed increased activation in the right amygdala compared to healthy controls. FND patients have altered processing of emotional stimuli, with implications for emotional regulation and symptom perpetuation in FND. | [86] | |
| Li et al 2015  10.1038/srep11635 | 18 | PNES | Cohort | fMRI | The PNES patients showed significantly increased fractional amplitude of low-frequency fluctuations (fALFF) mainly in the dorsolateral prefrontal cortex (DLPFC), parietal cortices, and motor areas, as well as decreased fALFF in the triangular inferior frontal gyrus. | [76] | |
| Maurer et al 2016  10.1212/WNL.0000000000002940 | 35 | FMD | Cohort | fMRI | Compared to the healthy controls, patients with functional movement disorders showed decreased functional connectivity between the right TPJ and the right sensorimotor cortex, cerebellar vermis, bilateral supplementary motor area, and right insula. | [80] | |
| Monsa et al 2018  10.1111/ene.13613 | 15 | Conversion disorder | Cohort | fMRI | Examination of inter-connectivity between networks showed a structure of disturbed connectivity, which included decreased connectivity between the DMN and limbic/salience network, increased connectivity between the limbic/salience network and body-related temporo-parieto-occipital junction network, decreased connectivity between the temporo-parieto-occipital junction and memory-related medial temporal lobe, and decreased connectivity between the medial temporal lobe and sensorimotor network. | [89] | |
| Mueller et al 2022  https://doi.org/10.1016/j.nicl.2022.102981 | 48 | Functional movement disorder | Cohort | fMRI | Comparing patients with and without functional weakness showed significant network centrality differences in the left temporoparietal junction and precuneus. Patients with functional weakness showed increased centrality in the same anatomical regions when comparing functional weakness with healthy controls. Moreover, in the same regions, patients with functional weakness showed a positive correlation between motor symptom severity and network centrality | [78] | |
| Piramide et al 2022  10.1007/s00415-021-10879-x | 40 | FMD | Cohort | fMRI | Compared to controls, FMD patients showed reduced FC between left M1 and left dorsal anterior cingulate cortex, and between right M1 and left M1, premotor/supplementary motor area (SMA), dorsal posterior cingulate cortex (PCC), and bilateral precuneus. | [81] | |
| Sojka et al 2019  10.3389/fneur.2019.00861 | 15 | FND | Cohort | fMRI | FND patients showed increased activation in several brain areas when observing negative pictures, notably in the post-central gyrus, precuneus, posterior cingulate cortex (PCC) and cerebellar vermis, and also in their emotion regulation condition, particularly in the precuneus and post-central gyrus. | [82] | |
| Szaflarski et al 2022  10.1016/j.yebeh.2022.108712 | 52 | Functional Seizures | Cohort | fMRI | fMRI activation to sad faces in the bilateral posterior cingulate cortex (PCC) and to neutral faces in the right anterior insula. Within-group linear regression revealed that with increasing DD, there was increased fMRI activation to sad faces in the PCC and to happy faces in the right anterior insula/inferior frontal gyrus (AI/IFG). | [71] | |
| van der Kruijs et al 2012  10.1136/jnnp-2011-300776 | 11 | PNES | Cohort | fMRI | For PNES patients, stronger connectivity values between areas involved in emotion (insula), executive control (inferior frontal gyrus and parietal cortex) and movement (precentral sulcus) were observed, which were significantly associated with dissociation scores. | [60] | |
| van der Kruijs et al 2014  10.1016/j.jpsychires.2014.03.010 | 21 | PNES | Cohort | fMRI | Patients displayed higher dissociation scores, lower cognitive performance and increased contribution of the orbitofrontal, insular and subcallosal cortex in the fronto-parietal network; the cingulate and insular cortex in the executive control network; the cingulate gyrus, superior parietal lobe, pre- and postcentral gyri and supplemental motor cortex in the sensorimotor network; and the precuneus and (para-) cingulate gyri in the default-mode network. | [62] | |
| Voon et al 2011  10.1002/mds.23890 | 11 | Conversion disorder | Cohort | fMRI | During both internally and externally generated movement, conversion disorder patients relative to normal volunteers had lower left supplementary motor area (SMA) (implicated in motor initiation) and higher right amygdala, left anterior insula, and bilateral posterior cingulate activity (implicated in assigning emotional salience). These findings were confirmed in a subgroup analysis of patients with tremor symptoms. | [66] | |
| Voon et al 2010  10.1093/brain/awq054 | 16 | Conversion disorder | Cohort | fMRI | Post hoc analyses revealed that whereas healthy volunteers had greater right amygdala activity to fearful versus neutral compared with happy versus neutral as expected, there were no valence differences in patients with conversion disorder. There were no group differences observed. | [147] | |
| Wegrzyk et al 2017  10.1016/j.nicl.2017.10.012 | 23 | FND | Cohort | fMRI | The most discriminative connections included regions such as the right caudate, amygdala, prefrontal, and sensorimotor regions, which were found to be hyperconnected in patients compared to controls. | [79] | |
| Weber et al 2024  10.1016/j.nicl.2024.103583 | 79 | FND | Case Control | fMRI | Insular co-(de)activation patterns related to the salience network, the somatomotor network and the default mode network were detected, which patients entered more frequently than controls. Moreover, an insular co-(de)activation pattern with subcortical regions together with a wide-spread co-(de)activation with diverse cortical networks was detected, which patients entered less frequently than controls. In patients, dynamic alterations conjointly correlated with amylase measures and duration of symptoms. | [70] | |
| **MEG** | | | | | | | |
| Boutros et al 2019  10.1177/1550059418792454 | 6 | psychogenic non-epileptic seizures (PNES) | Cohort | MEG | Analysis shows posterior-occipital alpha power to be decreased but fronto-temporal delta/theta power increased in people with PNES compared with HC subjects. Analyses of mean interregional functional connectivity of 54 brain regions, patients with PNES tended to have reduced mean coherence in extra-fronto-temporal regions (ex-FTRs) while increased mean coherence in fronto-temporal regions (FTRs) compared with HC | [50] | |
| Fiess et al 2016  10.1016/j.jpsychores.2016.10.007 | 21 | FND | Cohort | MEG | Similar activity modulation by emotional picture category in patients with FND and HC suggests that the fast, automatic detection of emotional salience is unchanged in patients with FND, but involves an emotion-processing network spanning posterior and sensorimotor areas. | [51] | |
| **PET** |  |  |  |  |  |  | |
| Arthuis et al 2015  10.1136/jnnp-2014-309390 | 16 | psychogenic non-epileptic seizures (PNES) | Cohort | PET | In comparison to group analysis of healthy participants, the group analysis of patients with PNES exhibited significant PET hypometabolism within the right inferior parietal and central region, and within the bilateral anterior cingulate cortex. | [116] | |
| Tatli et al 2024  10.3389/fpsyt.2024.1336881 | 20 | Conversion disorder | Cohort | PET | When compared with the control group, statistically significant differences in z-scores were observed among all brain regions except for parietal superior R and cerebellum. No correlation was observed between the metabolisms of the left ACC and left medial PFC; left ACC and left temporal lateral cortex; cerebellum and left parietal inferior cortex despite the presence of positive correlations between these regions. | [117] | |
| **SPECT** | | | | | | | |
| Biraben et al 1999  https://doi.org/10.1684/j.1950-6945.1999.tb00294.x | 3 | PNES | Cohort | SPECT | SPECT is not a primary tool for diagnosis of pseudoseizures, but when patients undergoing presurgical investigation are injected during pseudoseizures, then SPECT is unlikely to show misleading perfusion changes due to activation effects | [130] | |
| Czarnecki et al 2011  10.1016/j.parkreldis.2011.01.012 | 10 | psychogenic tremor (PT) from essential tremor (ET). | Cohort | SPECT | In ET, rest imaging revealed increased rCBF (relative cerebral blood flow) in cerebellar hemispheres and left inferior frontal gyrus. During the motor task, ET patients demonstrated increased rCBF in the supplementary motor area (SMA) and contralateral motor cortex and reduced rCBF in the cerebellum and visual cortex. | [133] | |
| Damian et al 2021  10.1016/j.seizure.2021.02.030 | 10 | PNES | Cohort | SPECT | Ictal SPECT in FS patients have shown decreased perfusion in the posterior parietal cortex without significant increase in regional cerebral blood flow during non-epileptic attack | [118] | |
| Gallucci-Neto et al 2020  10.1016/j.psym.2020.05.016 | 26 | psychogenic non-epileptic seizures (PNES) | Cohort | SPECT | In PNES patients compared with temporal lobe epilepsy group, we found a consistent increase in regional cerebral blood flow in the right precuneus (Brodmann area 7; P = 0.003) and right posterior cingulate cortex (Brodmann area 31; P = 0.001), as well as a decrease in regional cerebral blood flow in the right amygdala (P = 0.027). | [131] | |
| Neiman et al 2009  10.1016/j.yebeh.2009.02.042 | 13 | PNES | Cohort | SPECT | Patients with epilepsy had 80% hypoperfusion in the epileptogenic zone, only 15-30% of FS patients showed ictal SPECT abnormalities, mainly in bifrontal, left frontoparietal, right medial temporal, and right insular areas | [128] | |
| Olver et al 2019  https://doi.org/10.1136/jnnp-2018-320173 | 6 | PNES | Cohort | SPECT | increased co-activation of right ventromedial prefrontal cortex and right insula | [146] | |
| Varma et al 1996  10.1111/j.1600-0404.1996.tb07035.x | 10 | PNES | Cohort | SPECT | normal SPECT scans might support a diagnosis of PNES, focal hypoperfusion similar to that seen in epilepsy can occasionally be observed in patients with PNES | [127] | |
| Vuilleumier et al 2001  10.1093/brain/124.6.1077 | 7 | Conversion disorder | Cohort | SPECT | consistent decrease of regional cerebral blood flow in the thalamus and basal ganglia contralateral to the deficit. Independent parametric mapping and principal component statistical analyses converged to show that such subcortical asymmetries were present in each subject. Importantly, contralateral basal ganglia and thalamic hypoactivation resolved after recovery. Furthermore, lower activation in contralateral caudate during hysterical conversion symptoms predicted poor recovery at follow up. | [134] | |
| Yaźići et al 1998  10.1016/s0925-4927(98)00039-0 | 5 | Conversion disoder | Cohort | SPECT | Uptake ratios between areas of decreased perfusion and normal brain regions were considered significantly decreased when there was a change > or = 10%. Four of the five patients had left temporal and one patient had left parietal perfusion decreases. Uptake ratios ranged from 0.72 to 0.88 (mean+/-S.D.: 0.81+/-0.08). Our findings suggest that alterations in regional brain perfusion may accompany conversion symptoms | [132] | |

Table 3: Studies in Chronic Pain

| Study | Sample size | Patients included – Type | Study design | Methodology | Results | References |  |
| --- | --- | --- | --- | --- | --- | --- | --- |
| **EEG** | | | | | | |  |
| Babiloni et al 2006  10.1016/j.jpain.2006.03.005. | 10 | Chronic back pain | Cohort | EEG | Results showed maximum (negative) correlations between the alpha 2 and alpha 3 ERD amplitude at the left central area and the subjective evaluation of pain intensity (P < .001). The stronger the anticipatory alpha 2 and alpha 3 ERD, the higher the subjective evaluation of pain intensity. | [40] |  |
| Boord et al 2008  10.1038/sj.sc.3102077 | 8 | Chronic pain | Cohort | EEG | Patients with neuropathic spinal cord injury (SCI) pain had significantly reduced EEG spectral reactivity in response to increased or decreased sensory input flowing into the thalamocortical network and decreased alpha wave power. | [34] |  |
| Day et al 2021  10.1093/pm/pnab049 | 57 | chronic low back pain (CLBP) | Prospective | EEG  cognitive therapy (CT), mindfulness-meditation (MM), and mindfulness-based cognitive therapy (MBCT) | A significant reduction in theta (P=.015) and alpha (P=.006) power in the left frontal ROI across all treatments was found, although change in theta and alpha power in this region was not differentially associated with outcome across treatments. There were significant reductions in beta power in all five ROIs across all treatments (P≤.013). Beta power reduction in the central ROI showed a significant association with reduced pain intensity in MBCT only (P=.028). | [42] |  |
| De Blasio et al 2023  10.1016/j.clinph.2023.02.173 | 20 | endometriosis-related chronic pelvic pain | Cohort | EEG | Relative to controls, the endometriosis group had greater component amplitudes in delta (0.5 Hz) and beta (∼28 Hz), and reduced alpha (∼10 Hz). Delta and beta amplitudes were positively associated with pain severity, but only beta maintained this association after delta-beta amplitude coupling was controlled. | [44] |  |
| Fallon et al 2018  10.1002/ejp.1076 | 19 | Fibromyalgia | Cross-sectional study | EEG | FM patients exhibited greater pain, tiredness and tension on the day of testing relative to healthy control participants and augmented theta activity in prefrontal and anterior cingulate cortices. No significant differences were seen in other frequency bands. Augmented frontal theta activity in FM patients significantly correlated with measures of tenderness and mean tiredness scores. | [47] |  |
| Jensen et al 2013  10.1038/sc.2012.84 | 38 | Chronic pain | Cross-sectional study | EEG | Few significant associations between pain severity and EEG activity measures activity were found, more alpha activity associated with more pain as measured from frontal electrode sites. | [35] |  |
| Makowka et al 2023  https://dx.doi.org/10.1371/journal.pone.0281986 | 16 | Fibromyalgia | Cross-sectional | EEG | FM patients displayed lower functional connectivity in the High beta (Hbeta, 20-30 Hz) sub-band than controls (p = 0.039) in the left basolateral complex of the amygdala (p = 0.039) within the left mesiotemporal area, in particular, in correlation with a higher affective pain component level (r = 0.50, p = 0.049). Patients showed higher Low beta (Lbeta, 13-20 Hz) relative power than controls in the left prefrontal cortex (p = 0.001), correlated with ongoing pain intensity (r = 0.54, p = 0.032). | [48] |  |
| Sarnthein et al 2006  https://doi.org/10.1093/BRAIN/AWH631 | 15 | Chronic pain | Prospective | EEG | The average EEG power of all seven patients gradually decreased in the theta band and approached normal values only after 12 months. The excess theta EEG power in patients and its decrease after thalamic surgery suggests that both EEG and neurogenic pain are determined by tightly coupled thalamocortical loops. | [45] |  |
| Teixeira et al 2022  https://dx.doi.org/10.1093/pm/pnab293 | 30 | Chronic back pain | Cross-sectional study | EEG | Relative power of EEG in the beta, delta and theta bands as recorded from the frontal, central, and parietal cortical areas were significantly associated with Chronic pain modulation. | [41] |  |
| **fMRI** | | | | | | |  |
| Baliki  et al 2008  10.1523/JNEUROSCI.4123-07.2008 | 15 | Chronic back pain | Cohort | fMRI | Studying with fMRI a group of chronic back pain (CBP) patients and healthy controls while executing a simple visual attention task, we discovered that CBP patients, despite performing the task equally well as controls, displayed reduced deactivation in several key DMN regions. | [102] |  |
| Baliki et al 2011  10.1523/JNEUROSCI.1984-11.2011 | 15 | Chronic back pain | Cohort | fMRI | In the patients a correlation analysis related the medial prefrontal cortex (mPFC) aberrant BOLD high-frequency dynamics to altered functional connectivity to pain signaling/modulating brain regions, thus linking BOLD frequency changes to function. | [145] |  |
| Berry et al 2020  10.1093/pm/pnaa178 | 20 | Chronic back pain | Cohort | fMRI | increased fMRI responses to pain anticipation were observed in the right dorsolateral prefrontal cortex (dlPFC) and ventral posterior cingulate cortex (vPCC), | [103] |  |
| Bosma et al 2016  https://dx.doi.org/10.1002/hbm.23106 | 14 | Fibromyalgia (FM) | Cohort | fMRI &  temporal summation of second pain (TSSP) | fMRI analyses of perceptually equal TSSP identified similar brain activity in control and FM subjects; however, multiple areas in the brainstem (rostral ventromedial medulla and periaqueductal grey region) and spinal cord (dorsal horn) exhibited greater activity in control subjects. | [93] |  |
| Burgmer et al 2009  10.1007/s00702-009-0339-1 | 33 | FM & Chronic rheumatoid arthritis (RA) pain | Cohort | fMRI | We observed a FM-unique temporal brain activation of the frontal cortex in patients with FM. Moreover, areas of the motor cortex and the cingulate cortex presented a FM-specific relation between brain activity during pain anticipation and the magnitude of the subsequent pain experience. | [99] |  |
| Čeko et al 2020  10.1016/j.neuroimage.2020.116877 | 16 | Fibromyalgia | Cohort | fMRI | FM patients with current clinical pain during the scan had significantly increased default mode network (DMN) connectivity to bilateral anterior insula (INS) similar to previous studies. | [95] |  |
| Craggs et al 2012  10.1016/j.jpain.2012.01.002 | 13 | Fibromyalgia | Cohort | fMRI &  temporal summation of second pain (TSSP) | The models of effective connectivity were not identical in regions of the brain including thalamus, posterior insula and anterior midcingulate cortex (aMCC) within the left and right hemispheres between the FM and control groups, but were very similar. | [112] |  |
| Dammann et al 2020  10.1016/j.bbr.2019.112327 | 9 | craniomandibular disorder (CMD) | Prospective | fMRI & State -Trait Anxiety Inventory (STAI) | Reduction in STAI was associated with anterior insular fMRI-activation reduction on both hemispheres. We conclude that the anxiety driven anticipation of pain related to occlusal trigger is processed in the anterior insula and might therefore be a main driver of therapeutic intervention by the splint therapy in CMD. | [100] |  |
| DiMarzio et al 2019  10.1093/neuros/nyz269 | 15 | Chronic Pain | Cohort | fMRI & DBS | DBS resulted in more activity in anterior cingulate cortex (ACC) (P < 0.05) | [101] |  |
| Di Pietro et al 2020  10.1002/hbm.25087 | 15 | Complex regional pain syndrome (CRPS) | Cohort | fMRI | we found CRPS was associated with increases in resting signal intensity infra-slow oscillations (ISOs) (0.03-0.06 Hz) in the thalamus contralateral to the painful limb in CRPS subjects. CRPS subjects displayed stronger thalamo- somatosensory cortex functional connectivity than controls, and this was related to pain | [114] |  |
| Duke Han et al 2013  10.1002/gps.3898 | 64 | Chronic pain | Cohort | fMRI | Older adults with chronic pain showed greater functional connectivity between the posterior cingulate and left insula, left superior temporal gyrus, and left cerebellum. | [108] |  |
| Flodin et al 2014  10.1089/brain.2014.0274 | 17 | Fibromyalgia | Cohort | fMRI | FM patients showed a decreased connectivity between the right insula and a cluster of activity that covered the left primary sensorimotor areas. Moreover, a reduced degree of connectivity was observed for the right supramarginal gyrus and left inferior prefrontal cortex (PFC), and between thalamus and medial premotor cortex. | [104] |  |
| Ge et al 2021  https://dx.doi.org/10.1155/2021/6690414 | 18 | Chronic prostatitis/chronic pelvic pain syndrome (CP/CPPS) | Cross sectional study | fMRI | Compared with healthy control, the functional connectivity strength between left medial prefrontal cortex (mPFC) and posterior default mode network (DMN) decreased in the group of CP/CPPS | [107] |  |
| Hotta et al 2017  10.1016/j.jpain.2016.10.017 | 13 | complex regional pain syndrome (CRPS) | Cohort | fMRI | Brain areas with statistically significant group differences (q < .05, false discovery rate-corrected) included the hand representation area in the sensorimotor cortex, inferior frontal gyrus, secondary somatosensory cortex, inferior parietal lobule, orbitofrontal cortex, and thalamus. | [115] |  |
| Ichesco et al 2014  10.1016/j.jpain.2014.04.007 | 18 | Fibromyalgia | Cohort | fMRI | Healthy controls displayed greater connectivity between left anterior insular cortex and bilateral medial frontal gyrus/anterior cingulate cortex; and left posterior insular cortex and right superior frontal gyrus. Within the FM group, greater connectivity between the insular cortex and Cingulate cortex was associated with decreased pressure-pain thresholds. | [110] |  |
| Ichesco et al 2016  10.1002/ejp.832 | 12 | Fibromyalgia | Cohort | fMRI & pressure stimuli | Acute pressure pain increased insula connectivity to the anterior cingulate and the hippocampus. Additionally, we observed increased thalamic connectivity to the precuneus/posterior cingulate cortex, a known part of the default mode network, in patients but not in controls. | [109] |  |
| Jones et al 2021  https://dx.doi.org/10.1111/ner.13247 | | 5 | Poststroke pain | Cohort | fMRI & DBS | In response to pain, patients in the DBS OFF state showed significant activation (p < 0.001) in the same regions as healthy controls (thalamus, insula, and operculum) and in additional regions (orbitofrontal and superior convexity cortical areas). DBS significantly reduced activation of these additional regions and introduced foci of significant inhibitory activation (p < 0.001). | [113] |
| Kucyi et al 2014  10.1523/jneurosci.5055-13.2014 | 17 | Chronic temporomandibular disorder | Cohort | fMRI | Compared with healthy controls, we found that TMD patients exhibited enhanced mPFC functional connectivity with other DMN regions, including the posterior cingulate cortex (PCC)/precuneus (PCu) and retrosplenial cortex. | [96] |  |
| Lopez-sola et al 2014  https://dx.doi.org/10.1002/art.38781 | 35 | Fibromyalgia | Cohort | fMRI | fMRI revealed that patients showed reduced task-evoked activation in primary/secondary visual and auditory areas and augmented responses in the insula and anterior lingual gyrus. | [94] |  |
| Mandloi et al 2023  https://dx.doi.org/10.1111/jon.13117 | 7 | Chronic back pain | Cohort | fMRI | There were significant differences in FC of the insula between participants with chronic pain compared with HC. In the participants with pain, there was hyperconnectivity of the Anterior and posterior insula to the frontal pole. In addition, there was increased FC noted between the PI and the anterior cingulate cortex. Hyperconnectivity was also observed between the AI and the occipital cortex. | [106] |  |
| Mosch et al 2023  10.1016/j.nicl.2023.103355 | 23 | Fibromyalgia | Cohort | fMRI | Contrary to HC, FM failed to activate brain areas usually involved in pain modulation as well as reappraisal processes (right ventrolateral (VLPFC), dorsolateral prefrontal cortex (DLPFC) and dorsal anterior cingulate cortex (dACC)). | [111] |  |
| Müller et al 2021  10.1371/journal.pone.0235879 | 32 | Fibromyalgia | Cross-sectional | fMRI | We found no differences between cases and controls in resting-state cerebral blood flow of the thalamus, the basal ganglia, the insula, the somatosensory cortex, the prefrontal cortex, the anterior cingulum and supplementary motor area as brain areas previously identified to be involved in acute processing in fibromyalgia. | [97] |  |
| Napadow et al 2010  10.1002/art.27497 | 18 | Fibromyalgia | Cohort | fMRI | Patients with FM had greater connectivity within the DMN and right executive attention network (corrected P [P(corr)] < 0.05 versus controls), and greater connectivity between the DMN and the insular cortex. | [92] |  |
| Truini et al 2016  https://europepmc.org/article/med/27157397 | 20 | Fibromyalgia | Cohort | fMRI | Compared with control subjects, we identified that patients with fibromyalgia had an increased periaqueductal gray (PAG) connectivity with insula, anterior cingulate cortex, and anterior prefrontal cortex. The functional connectivity between PAG and the rostral ventral medulla, however, was not concordantly increased. | [98] |  |
| Tu et al 2019  10.1097/j.pain.0000000000001507 | 50 | Chronic low back pain (cLBP) | Cohort | fMRI | Results showed that the medial prefrontal cortex/rostral anterior cingulate cortex had abnormal FCs with brain regions within the default mode network and with other brain networks in cLBP patients. | [105] |  |
| Vartiainen et al 2009  10.1016/j.jpain.2009.02.006 | 8 | complex regional pain syndrome (CRPS) | Cohort | fMRI | In the patients, the distance between the thumb (D1) and little finger (D5) representations in primary somatosensory cortex was statistically significantly smaller in the hemisphere contralateral to painful side than in the hemisphere contralateral to healthy side. In the control subjects, the D1-D5 distance was the same in both hemispheres. | [91] |  |
| **MEG** | | | | | | |  |
| Alonso et al 2010  https://dx.doi.org/10.1007/s00221-010-2291-6 | 8 | Chronic temporomandibular disorder | Cohort | MEG | Specifically, Equivalent current dipoles (ECD) duration was longer in the TMD group in the precentral gyrus, and ECD onset time was earlier in the parietal operculum. | [59] |  |
| Choe et al 2018  10.1038/s41598-017-18999-z | 18 | Fibromyalgia | Cohort | MEG | In theta band, the slope of decrease in the number of connected components in barcodes showed steeper in contol, suggesting FM patients had decreased global connectivity. FM patients had reduced connectivity within default mode network, between middle/inferior temporal gyrus and visual cortex. | [56] |  |
| Gopalakrishnan et al 2016  10.1152/jn.00215.2016 | 9 | Central Poststroke pain | Cohort | MEG | Significantly greater responses were recorded in patients with pain (PS). PS exhibited significant parietal and frontal cortical activations in the beta and gamma bands. | [58] |  |
| Hsiao et al 2017  10.1186/s10194-017-0799-x | 28 | Fibromyalgia | Cohort | MEG | Compared with controls patients with fibromyalgia had decreased insula-DMN connectivity at the theta band and and the bilateral insula-DMN connectivity at the delta band (1-4 Hz) was negatively correlated with FM Symptom Severity. | [55] |  |
| Iwatsuki et al 2012  10.1016/j.ibneur.2021.05.001 | 21 | Complex Regional pain syndrome | Cohort | MEG | The amplitude envelope correlation (AEC) and Visual analogue scale (VAS) values were significantly correlated for the secondary (SII) somatosensory cortex and the precuneus and for the SII and insular cortex in the alpha frequency band in the right hemisphere. In the theta frequency band, the AEC and VAS values correlated for the SII and posterior cingulate cortex in the right hemisphere | [57] |  |
| Kisler et al 2020  10.1016/j.nicl.2020.102241 | 45 | Chronic back pain | Cohort | MEG | Compared to the healthy controls, the Chronic back pain patients exhibited increased theta power in the default mode (DMN) and decreased low-gamma power in the DMN and ascending nociceptive pathway (ANP), but did not exhibit beta-band attenuation or peak-alpha slowing. | [53] |  |
| Lim et al 2016  https://doi.org/10.3389/FNHUM.2016.00111 | 18 | Fibromyalgia | Cohort | MEG | The most remarkable finding was that FM patients had general increases in theta, beta and gamma power along with a slowing of the dominant alpha peak. Increased spectral powers in the theta-band were primarily localized to the left dorsolateral prefrontal (DLPFC) and orbitofrontal cortex (OFC). Beta and gamma over-activation were localized to insular, primary motor and primary and secondary somatosensory (S2) cortices, as well as the DLPFC and OFC. | [46] |  |
| Witjes et al 2021  https://doi.org/10.1097/PR9.0000000000000928 | 21 | Chronic pain | Cohort | MEG | The alpha power ratio was significantly higher (P < 0.05) in patients with chronic pain at both the sensor and brain source levels. The brain regions showing significantly higher ratios included the occipital, parietal, temporal and frontal lobe areas, insular and cingulate cortex, and right thalamus. | [52] |  |
| **PET** | | | | | | |  |
| Buvanendran et al 2010  https://pubmed.ncbi.nlm.nih.gov/20859325/ | 11 | Chronic cancer pain | Prospective | PET | Compared to patients with no pain, patients with moderate to severe pain had increased glucose metabolism bilaterally in the prefrontal cortex, BA 9-11. Unilateral activation was found in the right parietal precuneus cortex, BA 7. There were no areas of the brain in which there was decreased activity due to moderate to severe pain. | [123] |  |
| Chen et al 2007  https://pubmed.ncbi.nlm.nih.gov/18706225/ | 6 | Chronic brachial plexus avulsion | Prospective | PET | Compared with healthy subjects, the patients had significant glucose metabolism decreases in the right thalamus and SI (P < 0.001, uncorrected), and significant glucose metabolism increases in the right orbitofrontal cortex (OFC) (BA11), left rostral insula cortex and left dorsolateral prefrontal cortex (DLPFC) (BA10/46) (P < 0.001, uncorrected). | [122] |  |
| Harris et al 2007  10.1523/JNEUROSCI.2849-07.2007 | 17 | Fibromyalgia (FM) | Cohort | PET | FM patients display reduced MOR binding potential (BP) within several regions known to play a role in pain modulation, including the nucleus accumbens, the amygdala, and the dorsal cingulate. MOR BP in the accumbens of FM patients was negatively correlated with affective pain ratings. Moreover, MOR BP throughout the cingulate and the striatum was also negatively correlated with the relative amount of affective pain | [124] |  |
| Jääskeläinen et al 2001  https://doi.org/10.1016/S0304-3959(00)00409-7 | 10 | Burning mouth syndrome (BMS) | Cross-sectional study | PET | Significantly decreased presynaptic dopaminergic function in the putamen. | [126] |  |
| Jeon et al 2017  10.1097/md.0000000000005735 | 11 | Complex regional pain syndrome (CRPS) | Cohort | PET | The distribution volume ratio of [C]-(R)-PK11195 in the caudate nucleus (t(21) = -3.209, P = 0.004), putamen (t(21) = -2.492, P = 0.022), nucleus accumbens (t(21) = -2.218, P = 0.040), and thalamus (t(21) = -2.395, P = 0.026) were significantly higher in CRPS patients than in healthy controls. In patients with CRPS, there was a positive correlation between the DVR of [C]-(R)-PK11195 in the caudate nucleus and the pain score. | [120] |  |
| Martikainen et al 2015  10.1523/JNEUROSCI.4605-14.2015 | 16 | Chronic Non-neuropathic back pain (CNBP) | Cohort | PET | Patients with CNBP show reductions in dopamine D2/D3 receptor (D2/D3R) availability in the ventral striatum, linked to positive affect, pain tolerance, and affective pain components. During acute pain challenges, CNBP patients exhibit diminished dopamine release compared to controls. | [125] |  |
| **SPECT** | | | | | | |  |
| Chen et al 2007  10.1080/03009740601153790 | 71 | Fibromyalgia | Cohort | SPECT | The most prominent regional cerebral blood flow (rCBF) hypoperfusive region in both primary and concomitant FM groups was the left temporoparietal area, followed by the thalamus, right temporoparietal, frontal, and basal ganglia areas. Differences in rCBF hypoperfusion in these areas for both FM groups were not significant (all p>0.5). | [140] |  |
| Guedj et al 2007  10.1007/s00259-006-0174-7 | 18 | Fibromyalgia | Cohort | SPECT | Compared with control subjects, we observed individual brain SPECT abnormalities in FM patients, confirmed by SPM2 analysis, with hyperperfusion of the somatosensory cortex and hypoperfusion of the frontal, cingulate, medial temporal and cerebellar cortices. | [142] |  |
| Guedj et al 2008  10.2967/jnumed.108.053264 | 20 | Fibromyalgia | Cohort | SPECT | Fibromyalgia Impact Questionnaire (FIQ) total score was positively correlated with bilateral parietal perfusion, including postcentral cortex. These clusters of correlation were included in the areas of significant hyperperfusion. FIQ total score was also negatively correlated with perfusion of a left anterior temporal cluster, included in the areas of significant hypoperfusions. | [143] |  |
| Honda et al 2007  10.2302/kjm.56.48 | 15 | Chronic pain | Cohort | SPECT | The dorsolateral prefrontal area (both sides, right dominant), medial prefrontal area (both sides), dorsal aspect of the anterior cingulate gyrus nociceptive cortex (both sides) and the lateral part of the orbitofrontal cortex (right side) were found to have blood flow reduction in the group of patients with chronic pain. | [135] |  |
| Karibe et al 2010  10.1111/j.1440-1819.2010.02119.x | 10 | somatoform pain disorder | Cohort | SPECT | The patient group showed higher rCBF in the subcortical area, especially in the thalamus and cingulate gyri, than the control group. In contrast, the patient group showed lower rCBF in the bilateral frontal and occipital lobes as well as in the left temporal lobe. | [139] |  |
| Nakamura et al 2014  10.1007/s00776-014-0534-2 | 7 | Chronic low back pain (CLBP) | Cohort | SPECT | The CLBP group showed significantly reduced blood flow in the bilateral prefrontal cortex of the frontal lobe and increased blood flow in the bilateral posterior lobe of the cerebellum. | [136] |  |
| Papassidero et al 2023  https://dx.doi.org/10.1136/rapm-2022-104047 | 13 | Chronic low back pain (CLBP) | Cohort | SPECT | After using gabapentin, SPECT showed an increase of rCBF in the bilateral anterior cingulate gyrus and a decrease of rCBF in periaqueductal gray matter. Non-responder patients with gabapentin showed a post-treatment decrease of rCBF in the paracentral lobule of the brain. | [137] |  |
| Sundstorm et al 2006  10.1007/s00586-005-0040-5 | 27 | chronic neck pain | Cohort | SPECT | The non-traumatic patients displayed rCBF changes in comparison with the whiplash group and the healthy control group. These changes included rCBF decreases in a right temporal region close to hippocampus, and increased rCBF in left insula. The whiplash group displayed no significant differences in rCBF in comparison with the healthy controls. | [141] |  |
| Usui et al 2010  https://doi.org/10.1186/AR2980 | 29 | Fibromyalgia | Cohort | SPECT | Compared to control subjects, we observed rCBF abnormalities in fibromyalgia including hypoperfusion in the left culmen and hyperperfusion in the right precentral gyrus, right posterior cingulate, right superior occipital gyrus, right cuneus, left inferior parietal lobule, right middle temporal gyrus, left postcentral gyrus, and left superior parietal lobule | [144] |  |
